# MR-guidance in clinical reality: current treatment challenges and future perspectives

**DOI:** 10.1186/s13014-019-1308-y

**Published:** 2019-06-03

**Authors:** S. Corradini, F. Alongi, N. Andratschke, C. Belka, L. Boldrini, F. Cellini, J. Debus, M. Guckenberger, J. Hörner-Rieber, F. J. Lagerwaard, R. Mazzola, M. A. Palacios, M. E. P. Philippens, C. P. J. Raaijmakers, C. H. J. Terhaard, V. Valentini, M. Niyazi

**Affiliations:** 1 Department of Radiation Oncology, University Hospital, LMU Munich, Marchioninistraße 15, 81377 Munich, Germany; 2 Department of Radiation Oncology, IRCSS Sacro Cuore don Calabria Hospital, Negrar-Verona, Italy; 3 University of Brescia, Brescia, Italy; 4 Department of Radiation Oncology, University Hospital Zürich, University of Zurich, Zürich, Switzerland; 5 Istituto di Radiologia, Università Cattolica del Sacro Cuore, Rome, Italy; 6 Dipartimento di Diagnostica per Immagini, Radioterapia Oncologica ed Ematologia, Fondazione Policlinico Universitario “A. Gemelli” IRCCS, UOC di Radioterapia Oncologica, Rome, Italy; 7 Department of Radiation Oncology, Heidelberg University Hospital, Heidelberg, Germany; 8 Heidelberg Institute of Radiation Oncology (HIRO), Heidelberg, Germany; 9 Clinical Cooperation Unit Radiation Oncology, German Cancer Research Center (DKFZ), Heidelberg, Germany; 10 Department of Radiation Oncology, VU medical center, Amsterdam, The Netherlands; 11 Department of Radiation Oncology, University Medical Center Utrecht, Utrecht, The Netherlands

**Keywords:** MR-guided radiotherapy, Image-guided, radiotherapy, MR-IGRT, MR-Linac, adaptive radiotherapy, Inter-fraction variability, Intra-fraction fraction variability, MRI, outcome

## Abstract

Magnetic Resonance-guided radiotherapy (MRgRT) marks the beginning of a new era. MR is a versatile and suitable imaging modality for radiotherapy, as it enables direct visualization of the tumor and the surrounding organs at risk. Moreover, MRgRT provides real-time imaging to characterize and eventually track anatomical motion. Nevertheless, the successful translation of new technologies into clinical practice remains challenging. To date, the initial availability of next-generation hybrid MR-linac (MRL) systems is still limited and therefore, the focus of the present preview was on the initial applicability in current clinical practice and on future perspectives of this new technology for different treatment sites.

MRgRT can be considered a groundbreaking new technology that is capable of creating new perspectives towards an individualized, patient-oriented planning and treatment approach, especially due to the ability to use daily online adaptation strategies. Furthermore, MRL systems overcome the limitations of conventional image-guided radiotherapy, especially in soft tissue, where target and organs at risk need accurate definition. Nevertheless, some concerns remain regarding the additional time needed to re-optimize dose distributions online, the reliability of the gating and tracking procedures and the interpretation of functional MR imaging markers and their potential changes during the course of treatment. Due to its continuous technological improvement and rapid clinical large-scale application in several anatomical settings, further studies may confirm the potential disruptive role of MRgRT in the evolving oncological environment.

## Introduction

Advanced radiation techniques, including intensity modulated radiation therapy (IMRT), volumetric modulated arc therapy (VMAT) or high-dose stereotactic body radiotherapy (SBRT) pursue the goal of delivering high doses to the tumor, while sparing the surrounding tissues and organs at risk (OARs). To ensure a precise dose delivery, image-guided radiotherapy (IGRT) has been developed and widely introduced into clinical practice. Current IGRT techniques using on-board cone-beam CT (CBCT) are already very effective, but are limited due to the reduced soft-tissue contrast. Frequently, it remains challenging to distinguish tumor from normal tissues, with the consequence that dose escalation strategies are not readily feasible, or generous planning target volume (PTV) margins are applied to account for uncertainties in gross tumor volume (GTV) delineation, dose delivery and target coverage.

On-board real-time Magnetic Resonance Imaging (MRI)-guided radiotherapy (MRgRT) with hybrid MR-linear accelerator (MRL) systems marks the beginning of a new era. MRI is the most versatile and suitable imaging modality for RT, as it provides direct visualization of the tumor and surrounding tissue anatomy. Moreover, it provides real-time imaging to characterize and eventually track anatomical motion. Respiratory gating by MRI is particularly advantageous in several aspects for high dose SBRT [1, 2]. It enables motion mitigation and a reduction of PTV margins and allows for an accurate dose delivery to the PTV by reducing dose exposure of OARs. Certain anatomical sites or specific organs affected by motion from different sources (e.g. breathing, bowel displacement /bladder filling) may benefit from MR-guided gating techniques: thoracic tumors, including lung or mediastinal lesions, breast cancer, and abdominal or pelvic tumors, such as liver or pancreatic lesions and prostate cancer. Moreover, real-time plan adaptation, while the patient is on the treatment table, is a disruptive concept of the innovative MR-linear accelerator (MRL) workflow [3]. This new key feature will allow physicians to optimize dose escalation strategies, as there is a further potential for reducing dose to OARs, especially when a precise localization and real-time tracking of the tumor is ensured.

## Clinical sites

Successful translation of new technologies into clinical practice remains challenging. To date, the initial availability of next-generation hybrid MR-linac systems is still limited and therefore, the focus of the present preview is on the initial applicability in current clinical practice and on future perspectives of this new technology for different treatment sites.

### Brain

Tumors of the central nervous system (CNS) are frequently treated with RT. Specific entities are metastases, primary brain tumors (low-grade gliomas, anaplastic astrocytomas, oligodendrogliomas, glioblastomas), extra-axial tumors such as meningioma, and other benign entities including pituitary adenomas and vestibular schwannomas. A MRI-based planning workflow could potentially be both, cost- and time-saving while reducing uncertainties associated with CT-MRI registration [4]. MRI already represents the gold-standard imaging method for brain tumor diagnosis and the assessment of treatment response [5]. In this context, MRgRT allows for the first time to obtain both, structural and functional information during RT and to manage the adaptation of the prescribed dose during the treatment, in order to optimize outcome. To date, in daily clinical practice, a recent MRI is usually co-registered to bony structures of a simulation CT, achieving a high degree of confidence. Thus, due to these consolidated procedures, RT is already commonly delivered with a high level of precision to brain targets. Therefore, as well as hypothesized after the introduction of PET-MRI, a lot of concerns could be related to the real usefulness of MRgRT in brain RT.

However, a crucial difference emerges: the MRL systems enable a rapid adaptation, immediate target volume delineation [6] and quick tumor response assessment. An example is the treatment of a resection cavity, which can change significantly in shape and size between the simulation MRI and the initiation of treatment [4]. Furthermore, if hypofractionated stereotactic radiosurgery (SRS) is applied, the resection cavity could also change during the treatment course of 3–5 fractions, which would be visible using MRgRT. Tseng and colleagues assessed the dosimetric impact of the magnetic field, including the electron return effect at tissue-air boundaries in SRS and could show that neither target conformity nor dose gradient were negatively impacted [7]. Moreover, Wen and colleagues demonstrated, that excellent plan quality and dose delivery accuracy was achievable on the MRL system for treating multiple brain metastases with a single isocenter [8]. Besides high-dose fractionation schemes, it is expected that conventionally fractionated to moderately hypofractionated schedules will represent the standard-of-care in primary brain tumors due to improved therapeutic ratios. Nevertheless, it remains unknown, which advantages can result from the daily targeting and planning optimization by MRgRT, since the available MRI sequences, which are currently still very limited, may be improved in the future. To date, changes in gross tumor volume (GTV) [9] would at least allow early adaptation of the treatment plan.

In summary, MRgRT creates a new perspective towards an individualized, patient-centric planning approach using online adaptation for intracranial treatments. Furthermore, a significant increase in knowledge is expected concerning the biological processes, which occur during RT and its effect on patient survival for brain diseases.

### Head & Neck

**Fig. 1:**
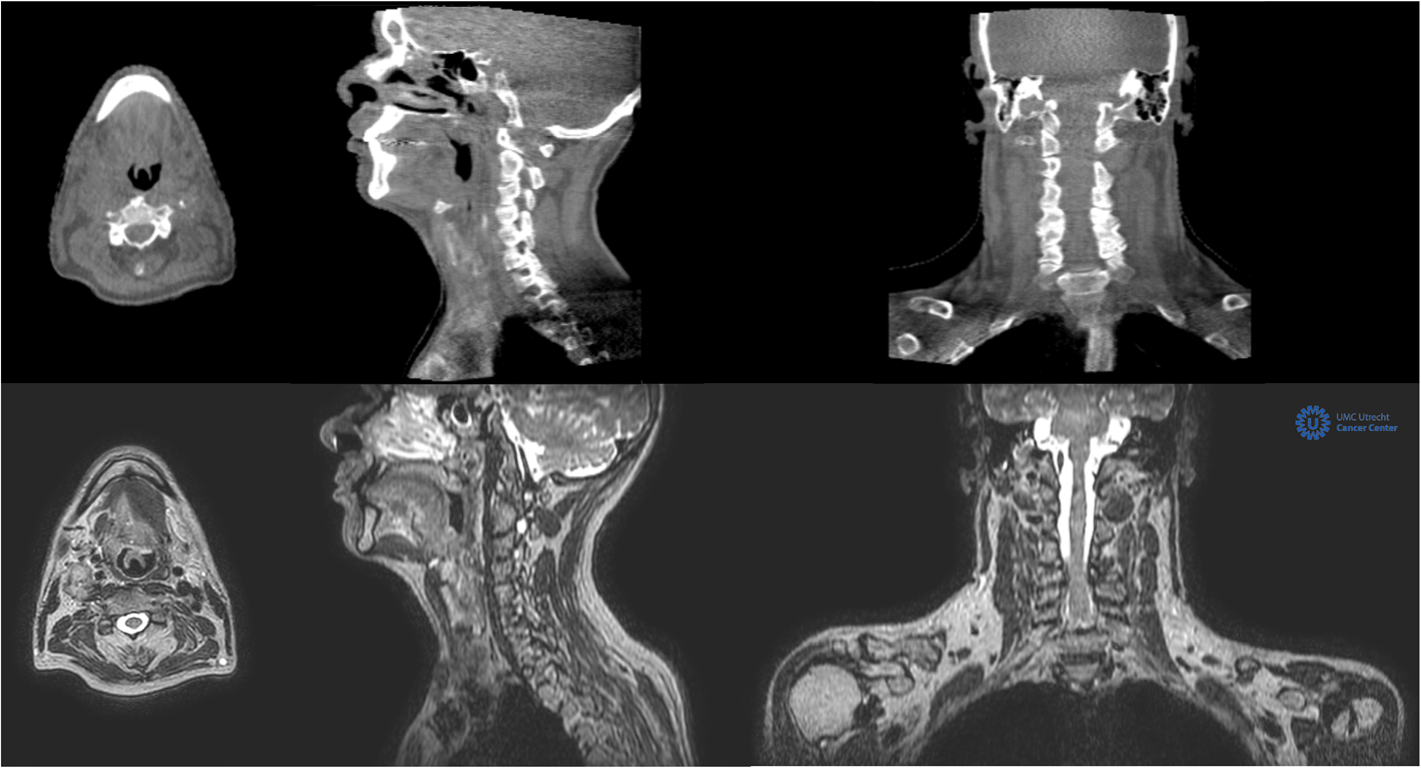
Cone beam CT images of an oropharyngeal cancer patient (upper row) compared to the 1.5 T MR images (T1 3D 0.7 × 0.7 × 1.2 mm^3^) of the same patient acquired at the MRL (lower row)

MRI is increasingly used in head and neck (H&N) RT due to its superior soft tissue contrast and its versatility. MRI is utilized in treatment planning to delineate the GTV [10], the clinical target volume (CTV) [11] and to estimate the necessary PTV margin [12] and to assess the loco-regional treatment response [13]. Undoubtedly, the advent of MRL [3] opens the door to fully exploit the advantages of MRI over CBCT by its online adaptation capability during the treatment procedures (Fig. 1). The following significant improvements are anticipated:

#### Adaptation to anatomical changes

During the course of irradiation, H&N patients can significantly lose weight and OARs, such as the parotid glands can dramatically shrink [14]. The time scale of these changes does not require online optimization. However, the MRL and its workflow are designed to inherently manage these potential changes and online, offline or weekly adaptation can be applied for optimal OAR sparing.

#### Adaptation to tumor response

Tumor response varies from significant volumetric changes of large lymph nodes to more subtle MR signal changes within the GTV. When the tumor clearly shrinks and is replaced by healthy tissue, the GTV might be adapted in a straightforward manner [15]. How to adapt to MR signal intensity changes within the initial GTV must be investigated in well-designed clinical trials. For oropharyngeal cancer, a distinction should be proposed between HPV positive patients, where dose de-escalation could be considered for well-responding GTVs, and HPV-negative patients that require dose escalation to poor-responding regions inside the GTV.

#### Motion management

H&N cancer patients are treated using an immobilization mask that, in combination with the several rigid bony structures, minimize major intrafraction motion. However, considerable motion has been observed for the larynx and the tongue due to breathing, movement of the tongue and swallowing [12]. Cine-MR during radiation can be applied to guarantee minimal PTV margins. Furthermore, exception gating might be applied to interrupt irradiation in case of excessive motion.

Curative treatment schemes for H&N cancer patients usually consist of 30 to 35 fractions. Full online plan optimization including the registration, adaptation, optimization and QA steps currently takes approximately 45 min [3] for relatively simple dose distributions. Nevertheless, thirty fractions of 45 min in a noisy, claustrophobic environment is probably too distressing for many patients. This discomfort might be reduced by developing a quick MRL workflow when minimal adaptation is needed, and apply full online adaptation only, when major changes occur. Furthermore, comfortable patient positioning methods including noise reduction will be developed. Both patient comfort and a reduced workflow are prerequisites to fully exploit the promises of MR-guidance for head-and-neck cancer patients.

### Lung

Non-small-cell lung cancer (NSCLC) histology accounts for approximately 85% of all lung cancer cases. Of these, almost 30% present with locally advanced disease, and RT in combination with chemotherapy represents the treatment of choice for this patient group [16–19]. Because of the low survival rates, dose escalation strategies for stage III NSCLC have been advocated [20, 21]. However, dose escalation for stage III NSCLC requires caution and should be thoroughly studied. Volumetric and positional changes throughout the course of RT have been reported, making adaptive irradiation for advanced lung cancer necessary in about 1/3 of the patients to ensure target coverage and reduce lung dose [22, 23]. Lung tumor motion is complex and is dependent on the location of the tumor in the lung and whether it is attached to rigid structures, such as the chest wall or vertebrae. Motion amplitudes of several centimeters have been reported in the literature [24]. By direct visualization of the “real-time” tumor position in combination with respiratory gated dose delivery, an MR-guided treatment unit can offer a much more accurate and precise dose delivery, without the use of any surrogate or statistical model for respiration [1, 25].

SBRT is a well-established technique for the management of stage I NSCLC, which has significantly improved local control (LC) in comparison to conventional fractionation. LC rates of ≥85% are achieved when the prescribed biologically equivalent tumor dose is ≥100 Gy [26–29]. It is common practice to generate treatment volumes for lung SBRT from 4D-CT acquisition [29, 30]. However, this can lead in some instances to large treatment volumes whereas MR-guided SBRT treatment delivery for lung tumors has shown promising results in terms of treatment volume reduction and intra-fraction motion management [1, 2]. SBRT has also been shown to be an effective modality for treating patients after failure of conventional irradiation and metastatic lung tumors, achieving good local control with acceptable toxicity [31–35]. Recent reports regarding online plan adaptation for SBRT treatments under MR-guidance have shown promising results [36–38]. A mid-treatment approach for plan adaptation for centrally located thoracic tumors allowed reduction of OAR violations and recovery of PTV coverage due to interfractional changes [39].

In summary, MgRT offers improved accuracy of the target position by means of superior intra-fraction tumor visualization. MRgRT is expected to achieve prolonged disease-free survival and lower toxicity for thoracic lung tumors, especially in the field of re-irradiation and in the management of centrally located lesions, by using better intra-fraction motion management in combination with online plan adaptation.

### Breast

The standard of care for patients with early breast cancer after breast conserving surgery is whole breast irradiation [40, 41]. Recently, new treatment approaches using partial breast irradiation (PBI) or accelerated partial breast irradiation (APBI) in low-risk tumors were analyzed [42]. PBI aims to reduce irradiated breast volume in order to decrease long-term side-effects of treatments, optimizing cosmetic outcomes and improving quality of life while maintaining local tumor control [43]. Nevertheless, conflicting results concerning toxicity and cosmetic outcome have been reported [44, 45].

A possible concern of the inconclusive data are differences in target volume delineation, the dosimetric characteristics and the dose-fractionation schedules of the various APBI techniques. Localization and delineation of the CTV on a postoperative CT remains difficult, even if additional clips are placed in the tumor bed. Furthermore, additional margins must be added to the CTV to account for chest wall movement and patient set-up in External Beam RT (EBRT), which may result in larger irradiated volumes in EBRT compared to brachytherapy or intra-operative APBI techniques [46, 47]. The challenge of adequate target definition in postoperative RT could be mastered with MRgRT, as MRI has excellent soft-tissue contrast, especially in the visualization of irregularities and spiculations [48].

Another approach could be the preoperative MRgRT APBI. Preoperative target delineation showed to have less inter-observer variation as compared to the postoperative setting [49, 50]. MRI has a high sensitivity for detection of invasive breast cancer and a good correlation with histopathology findings [48]. To date, different groups evaluated the concept of single dose APBI delivered prior to surgical resection and treated the first patients [51, 52]. Horton et al. [52] designed a phase I dose escalation trial of a single-dose preoperative radiation treatment for early stage breast cancer patients (node-negative, invasive breast cancer or DCIS ≤2 cm). There were three different dose escalation levels of 15 Gy (*n* = 8), 18 Gy (*n* = 8) or 21 Gy (*n* = 16) and lumpectomy was performed within 10 days. The CTV was delineated using a planning MRI, and included the GTV with an isotropic margin of 15 mm. Overall, no acute dose-limiting grade 3 radiation-related toxicities were reported. These early results seem encouraging and represent a first step toward a novel APBI approach [52].

In summary, set-up margins can be further reduced, as no co-registration of planning MRI and CT is required and dose delivery can be performed using respiratory gated MRgRT. This approach can reduce irradiated breast volume and therefore normal tissue toxicity, as cardiac toxicity [53, 54]. Moreover, MR-guided preoperative RT could potentially facilitate dose escalation and enable an ablative, definitive treatment approach for early-stage breast cancer. Obviously, the MRgRT approach for breast cancer needs to be tested in further clinical trials, but it already appears to have the potential to become a future “game changer” in the portfolio of individualized breast RT strategies.

### Gastrointestinal tumors

#### Liver

Liver represents an intriguing anatomic site of application for MRgRT SBRT due to the increasing utilization of MRI in the characterization of primary and secondary hepatic lesions and the emergent role of SBRT in their management [55, 56]. Kishan et al. [57] evaluated the dosimetric feasibility of Tri-Cobalt-60 MR-guided RT liver SBRT and observed optimal liver and kidney sparing, especially for the most peripheral lesions.

Furthermore, MRI real-time 2-Dimension gating imaging can efficaciously manage treatment volumes movements through direct and/or indirect gating approaches and overcome the necessity of invasive fiducials implantation [58]. Despite the promising technical solutions, the clinical evidence about liver MRgRT still remains anecdotal [59].

#### Pancreas

The anatomical characteristics and location of the pancreas make it difficult to find the balance between target coverage and OAR sparing, especially in the SBRT setting. Available technologies for patient re-positioning and dose delivery (CBCT, motion management solutions) do not allow effective dose escalation of the target and toxicity remains a strong dose-limiting factor [60–66].

Various studies have described the segmentation advantages and planning solutions for MRgRT in this scenario; in particular, its online adaptive approach, which appears suitable for dose escalation, plan adaptation and inter-fraction anatomical variability management [59, 67, 68]. Larger studies are needed to evaluate the occurrence of toxicity with this approach. Nevertheless, the first clinical results on a very limited number of patients seem promising [59, 69]. For these reasons, pancreatic cancer represents one of the most important applications of MR-guided RT and is a good candidate for further developments of online adaptive solutions.

#### Rectum

To date, MRI represents the gold standard technique in rectal cancer diagnosis, due to its excellent soft tissue contrast and high spatial resolution. The integration of this kind of imaging in hybrid MRgRT solutions opens up new frontiers for segmentation and dose escalation protocols [70]. Further advantages will come from the use of specific MRI sequences, such as diffusion weighted imaging (DWI), and radiomics applications throughout the course of RT treatment to identify new target volumes and assess or predict response [71, 72].

Clinical studies on rectal cancer MRgRT are not yet available in literature, but its feasibility and safety in the neoadjuvant setting have been evaluated. Treatment plans of the Tri-60-Co MRL systems reach comparable target coverage, although larger volumes of OARs (i.e. small bowel) receive higher low-moderate doses as compared to standard intensity-modulated RT technologies [73]. These results encourage MRgRT applications with higher energy systems (MRL) on large rectal cancer patient cohorts.

### Urogenital tumors

#### Kidney

Although renal cell carcinoma (RCC) has historically been considered a radioresistant entity, and RT has been usually applied with palliative intent, recent technological advancements are allowing radiation oncologists to introduce RT with a curative intent also in this setting. Ongoing studies confirming the safety and efficacy of preliminary reported data are likely to open a scenario, in the near future, that integrates SBRT into the therapeutic algorithm of primary RCC [74, 75].

Nevertheless, the kidney is affected by large intra-fraction respiratory variations that can dramatically change during the treatment of daily fractionation [76–80]. Stemkens et al. [81] developed a calculation method to evaluate the accumulated dose for MR-guided SBRT of RCC in case of intra-fraction respiratory modifications, determining the effect of such uncertainties on the deposited dose. In their small patient cohort, these variations showed large dosimetric differences with respect to the planned dose distribution, confirming the potential role of online MR-guidance combined with real-time treatment planning adaptation during daily SBRT delivery for RCC. Moreover, Stam and colleagues showed that the dosimetric feasibility of MRgRT was strictly related to the geometry of the affected kidney, the dimension of the target and the proximity of the bowel during the daily online evaluation. A maximum diameter of the kidney lesion of 35 mm was considered the cut-off for a safe treatment without violation of the OAR constraints [82].

In summary, considering the previous discussed uncertainties related to respiratory variations and the individual anatomy conformation of the region of interests, kidney tumor irradiation by MRL seems promising. MRgRT for primary and metastatic tumors in the kidney may represent a new tool to expand its therapeutic application in the near future, although it is still under development due to the paucity of available clinical data.

#### Prostate

RT has a well-defined role in the management of organ-confined prostate cancer and is considered a standard curative treatment option, especially in the era of dose escalation and hypofractionation by IMRT and IGRT, and more recently by means of SBRT [83]. Despite the routinely adoption of daily IGRT to compensate for inter-fractional variations, the intra-fractional motion of the prostate gland and OARs [84, 85] during irradiation continues to be challenging [86]. Peng et al. [87] showed that, when the baseline treatment plan is superimposed on daily CBCT scans, about one third of the sessions would require an online plan adaptation due to the differences between planned and delivered dose to the prostate target and OARs. Obviously, these discrepancies become more relevant when ultra-fractionated schedules are adopted [88]. MR-guided image guidance can offer improved anatomical definition compared to on-board CBCT [89] while reducing radiation exposure. Furthermore, real-time MR imaging during dose delivery is able to take into account not only the systematic anatomical variability of prostate swelling, but also random anatomical changes, such as inter/intra-fraction bladder and rectal filling, as well as independent variations and deformations of OARs.

In fact, the most interesting benefit in prostate cancer RT is undoubtedly represented by the ability to perform daily adaptive replanning. With conventional IGRT, there are no possibilities to compensate for the independent movements of the prostate volume. At the beginning of the treatment, RT can induce a volumetric increase of the prostate gland followed by a decrease towards the end of the treatment [84]. In case of extreme hypofractionated schedules, the swelling may even persist after the end of treatment [90]. Therefore, the online adaptive strategies used by the MRL systems are likely to radically change the management of prostate cancer RT. Furthermore, online MR monitoring can automatically pause the treatment delivery if the prostate position exceeds a predefined threshold. Moreover, MRgRT enables to avoid specific radio-opaque markers that serve as a surrogate for the prostate position. Another clinical value that advocates MRgRT in prostate cancer is the role of predicting treatment response [91]. Specific MRI sequences could be used as an indicator for early tumor response, as confirmed by preliminary data on diffusion weighted imaging (DWI) during MRL delivery [91].

In summary, the recent developments of MRgRT systems open up new perspectives for RT in prostate cancer by enabling adaptive and on-line tracking strategies, especially when extremely high doses per fraction are prescribed. Furthermore, the capability to produce high quality MR sequences during and after the treatment, will probably further change the perspective of the MRI availability in this setting, opening an unexplored window on the landscape of radiomics for prostate cancer RT.

#### Bladder

Radical cystectomy and RT (with or without chemotherapy), are the two main treatment approaches for muscle-invasive bladder cancer [92]. Historically, RT has been reserved for patients with inoperable bladder tumors or when defined as medically unfit for cystectomy. A growing amount of evidence suggests that tri-modality treatment for bladder preservation is potentially able to obtain acceptable outcomes and can be considered a treatment option in selected patients [93, 94]. The tri-modality approach includes transurethral resection of the bladder cancer lesion followed by RT and concomitant chemotherapy.

However, one of the main criticisms regarding RT in bladder cancer is related to organ motion management. The bladder is a hollow mobile organ, seriously affected by changes in size and position during RT. This can dramatically impact daily dose coverage of the bladder tumor and OARs sparing, limiting the reliability and reproducibility of the entire RT [95–102]. To overcome this issue, large margins surrounding the target region are usually applied. Nevertheless, larger margins used to compensate uncertainties in treatment volume, result in increased toxicity [103, 104].

In order to check and correct the position, size and shape of the bladder for each treatment fraction, a high quality 3D image acquisition using CBCT has been introduced in clinical practice [105, 106]. Vestergaard and colleagues [107] tried to assess the optimal bladder target coverage by online MR-guided adaptive re-optimization using three kinds of margins: isotropic, anisotropic, and population-based. All three MR-guided adaptive strategies were able to obtain a large reduction in target volumes compared to a plan library approach. More specifically, the anisotropic margin resulted in the largest advantage in terms of PTV minimization [107]. This experience confirmed the promising role of MRL systems for online target shift check and correction during a treatment fraction for bladder cancer.

In summary, although some concerns remain in regard to the additional time needed to carry out online dose distribution re-optimization, the advent of MRL systems will undoubtedly improve bladder cancer adaptive RT strategies, reinforcing its indication in this setting [108].

### Gynecological tumors

Standard therapy for locally advanced cervical cancer is a combination of concurrent chemo-RT followed by brachytherapy [109]. Despite the wide application of daily image-guidance and advanced RT techniques including IMRT and VMAT, long-term urogenital and gastrointestinal side-effects are still frequent [110].

Due to its excellent soft-tissue contrast, MRI is already widely applied for staging and post-treatment evaluation of cervical cancer, as it is superior in assessing tumor size as well as soft tissue invasion compared to conventional CT imaging [111, 112]. However, regarding image-guidance, CBCT is still routinely used in RT, while MRI is recommended as the imaging method of choice for brachytherapy [113]. MR-guided brachytherapy is gradually becoming standard of care by allowing superior sparing of surrounding radiosensitive organs combined with dose escalation compared to conventional 2D-planning [114–117]. Based on the excellent results of MR-guidance in brachytherapy, it has been questioned for EBRT of cervical cancer, whether MRI could not only be applied for advanced tumor delineation but also for image-guidance [110, 114, 118]. The CTV for EBRT comprises the cervix and the uterus which are known to show significant inter- and intra-fractional motion due to the close proximity to hollow OARs [110, 119]. Large safety margins are usually needed in CBCT-imaged-guided RT to account for random and patient-specific organ movement [110, 119]. Due to the potential regression of cervical cancer of up to 60–80% of the pre-therapeutic tumor volume during EBRT, further pelvic organ motion might be expected during RT [118, 120].

MRgRT with its superior soft-tissue contrast allowing for precise and immediate detection of inter-fractional organ motion as well as tumor shrinkage in response to therapy includes the potential of reducing toxicity and potentiating dose escalation in EBRT for cervical cancer [110, 121]. Furthermore, functional MRI comprising non-invasive assessment of tissue perfusion, hypoxia or cellular density might be applied to guide RT treatment in cervical cancer with e.g. higher doses delivered to hypoxic tumor parts [110, 122–127]. While first shuttle-based approaches have shown that offline MRgRT is feasible for cervical cancer, the high potential of the new hybrid MRL devices is an immediate online adaptive treatment based on the anatomy of the day [3, 128–132]. Additionally, due to intra-fractional imaging, advanced motion management strategies, like gating become possible providing a “real-time” anatomical feedback with the advantage of further reducing safety margins [121]. A first case report about both, MR-guided EBRT and brachytherapy underlined the high potential of this new promising technique for cervical cancer [132].

In summary, MRg RT for cervical cancer can represent a promising tool to overcome the limits of conventional IGRT systems, in order to improve daily adaptive RT strategies. Further studies can confirm its potential disruptive role in this setting.

### Oligometastatic disease

Metastatic solid cancer was long considered incurable and treatment consisted mainly of palliative chemotherapy. Local treatments, such as surgery or radiotherapy, with palliative, non-ablative doses were restricted to symptom control. The concept of oligometastatic disease (OMD) is currently challenging this dogma by defining an intermediate state of metastasized disease, with a more favorable disease biology and dynamic. OMD is characterized by a limited number of metastatic lesions and a low overall metastatic burden that opens a therapeutic window for radical treatment to all metastatic sites. Originally coined by Hellman and Weichselbaum in 1995 [133], the idea has gained traction particularly during recent years through several developments: a) improved diagnostics for early detection of low disease burden b) clinical implementation of minimally invasive and high-precision locally-ablative treatments (LAT) such as video- or robotic assisted surgery (VATS, RATS) or SBRT c) more effective systemic treatments that have led to a prolonged overall survival (OS) of metastatic patients and d) a better biological and clinical understanding of tumor biology.

In the treatment of oligometastatic disease, early efforts have mainly focused on the radical treatment of readily resectable lesions, like brain and adrenal metastases. With the improvement in diagnostic imaging and novel developments in non-invasive LAT modalities such as SBRT, prospective reports have surfaced recently that investigate radical treatment of all disease sites, potentially leading to improved clinical outcome [134–136]. Still, a major concern is the potential toxicity from high local ablative radiotherapy dose, especially in anatomical regions not readily visualized with current IGRT methods (proximal bronchial tree, esophagus, duodenum, small and large bowel). The advent of MRgRT and the possibility to instantly adapt the RT dose to the daily anatomical situations open a window of opportunity to deliver high radiation doses while sparing surrounding normal tissue on a daily basis. In principle, all anatomical locations can be targeted in this way and most thoracic and abdominal indications have already been mentioned in this review. Therefore, we will focus our discussion on the advantages of MRgRT to the following clinical scenarios:

#### Lymph node metastases

In a recent review on SBRT for lymph node (LN) metastases, Jereczek-Fossa et al. reported local control rates of 64% up to 98% at 3 years [137]. A clear dose response correlation was observed as well. One of the latest reports could also correlate local control with overall survival [138]. Therefore, there is a relevant need to locally apply a sufficient dose in order to improve outcome. Depending on the visibility of lymph nodes in CBCT, this is difficult to achieve in certain cases and may even necessitate larger PTV margins to a certain proper targeting. A first MRI-guided planning approach to investigate the benefits of direct tumor visualization, margin reduction and improvement in dose delivery to OAR has been reported [139]. This technology improvement for better dose delivery is timely, as the interest in LN targeting especially in prostate cancer is becoming critical due to the outstanding detection rate of small LN metastases in PSMA PET [140]. As these targets are small, difficult to detect in CBCT, online MR-guidance is ideally suited to treat these lesions. It remains to be seen whether the first positive results of such an approach will translate into a durable clinical benefit [141].

#### Adrenal gland metastases

In the oligometastatic setting, radical treatment of adrenal metastases in the form of surgical resection is a well-established indication. Reports on CT guided SBRT have emerged with very encouraging local response rates, as long as the tumors can be readily visualized and a sufficient ablative radiation dose can be delivered [142, 143]. Local control rates of 32 to 90% have been reported with varying fractionation schedules. It is not surprising that this tumor site has been identified as a promising target for MRgRT, as more reliable visualization with online mitigation of tumor motion is possible. A first clinical report on MR-guided SBRT of adrenal glands showed significant inter-fraction displacements of OAR and the dosimetric benefit of online plan adaptation which resulted in consistently delivery of high radiation doses [37].

## Conclusions

In summary, MRgRT can be considered a groundbreaking new technology that is capable of creating new perspectives towards an individualized, patient-oriented planning and treatment approach, especially due to the ability to use daily online adaptation strategies. Furthermore, MRL systems overcome the limitations of conventional IGRT, especially in soft tissue, where target and OARs need accurate definition. Nevertheless, some concerns remain concerning the additional time needed to re-optimize dose distributions online, the reliability of the gating and tracking procedures and the interpretation of functional MR imaging markers and their potential changes during the course of treatment. Due to its continuous technological improvement and rapid clinical large-scale application in several anatomical settings, further studies may confirm the potential disruptive role of MRgRT in the evolving oncological environment.

## Data Availability

Not applicable.

## Abbreviations

APBI: Accelerated PBI
CBCT: Cone Beam Computed Tomography
CNS: Central nervous system
CT: Computed tomography
CTV: Clinical Target Volume
GTV: Gross Tumor Volume
IGRT: Image-Guided Radiotherapy
IMRT: intensity modulated radiation therapy
MRgRT: MRI guided-radiotherapy
MRI: Magnetic Resonance Imaging
MRL: MR-Linear accelerator
MVCT: Megavoltage computed tomography
OARs: Organs at risk
PBI: Partial Breast Irradiation
PET: Positron Emission Tomography
PTV: Planning Target Volume
RT: External Beam Radiation Therapy
SBRT: Stereotactic body radiotherapy
VMAT: volumetric modulated arc therapy
